# Pain Control in the African Context: the Ugandan introduction of affordable morphine to relieve suffering at the end of life

**DOI:** 10.1186/1747-5341-5-10

**Published:** 2010-07-08

**Authors:** Anne Merriman, Richard Harding

**Affiliations:** 1 Dept of International Programmes, Hospice Africa Uganda, Kampala, Uganda; 2 Department of Palliative Care, Policy & Rehabilitation, Cicely Saunders Institute, King's College, London, UK

## Abstract

Dr Anne Merriman is the founder of Hospice Africa and Hospice Africa Uganda. She is presently Director of Policy and International Programmes. Here she tells the story of how HAU was founded. Dr Richard Harding is an academic researcher working on palliative care in Sub-Saharan Africa. This paper described Dr Merriman's experience in pioneering palliative care provision. In particular it examines the steps to achieving wider availability of opioids for pain management for those with far advanced disease. Hospice Africa Uganda has been a model facility in achieving high quality clinical care embedded in a strategy of advocacy and education, using a multifaceted approach that has addressed logistical, policy and legislative barriers. Until 1990 control of severe pain in Sub-Saharan Africa was non-existent except in Zimbabwe and S Africa. Oral affordable morphine was brought to Kenya through Nairobi Hospice that year, and to Uganda through Hospice Africa Uganda in 1993. This paper offers an example of a highly effective and cost efficient model of care that has transformed the ability to humanely manage the problems of those with terminal illness, and to offer a culturally appropriate "good death". Thus it is now possible to complete the ethical circle of care in resource poor circumstances.

## Background

### Terminal disease: the need for palliative care

Sub-Saharan Africa faces a very high burden of incurable terminal disease. During 2007 there were 22.5 million people living with HIV infection; 1.7 million adults and children became infected with HIV; and 1.6 million died of AIDS (UNAIDS). The burden of cancer is just beginning to be understood and receive attention with clinical research and policy. The burden of other non-malignant diseases is unknown, although heart failure is recognized as a leading cause of death in Southern Africa. Palliative care can relieve the suffering of patients and families affected by these diseases and a strong body of evidence has demonstrated this [1]. The pain, symptoms, insight and anxiety associated with HIV can also be controlled under palliative care [2]. For this reason, policy and legal frameworks have sought to remove this unnecessary suffering and to promote cheap and effective palliative care as both a public health issue and as a human right [3]. Medical ethics requires that we "do no harm". Leaving my patient in pain in the era when pain relief and symptom control is affordable, is breaking our ethical code.

Since the modern palliative care speciality was developed and grew in the 1960's across Western Europe, Australia and North America it has (to a greater or lesser degree) become integrated into the health system. The palliative care needs of a range of patients and families can be met (although still only a proportion of those who could benefit actually get access). Therefore it is difficult to imagine, and impossible to justify, a country where there is no medicine for severe pain. This, however, is the case for many countries in Africa today. While there are centres of excellence providing care, education and advocacy, they are few and far between [4,5]. To reach the poorest and those suffering in an appropriate and ethical manner, a public health policy needs to be accepted and delivered in every country.

### What is palliative care?

WHO defines palliative care as follows [6]:

"Palliative care is an approach that improves the quality of life of patients and their families facing the problem associated with life-threatening illness, through the prevention and relief of suffering by means of early identification and impeccable assessment and treatment of pain and other problems, physical, psychosocial and spiritual. Palliative care:

• provides relief from pain and other distressing symptoms;

• affirms life and regards dying as a normal process;

• intends neither to hasten or postpone death;

• integrates the psychological and spiritual aspects of patient care;

• offers a support system to help patients live as actively as possible until death;

• offers a support system to help the family cope during the patients illness and in their own bereavement;

• uses a team approach to address the needs of patients and their families, including bereavement counselling, if indicated;

• will enhance quality of life, and may also positively influence the course of illness;

• is applicable early in the course of illness, in conjunction with other therapies that are intended to prolong life, such as chemotherapy or radiation therapy, and includes those investigations needed to better understand and manage distressing clinical complications."

In summary, palliative care is appropriate for any patient with an incurable, progressive and life threatening illness, and should be provided from the point of diagnosis until the end of life- and should provide bereavement support for family members if required. It is patient focused rather than disease oriented, holistic, and can be provided alongside potentially curative options. Indeed, palliative care is entirely necessary alongside antiretroviral therapy due to the distressing and complex symptoms experienced by those living with what remains an incurable terminal disease [7,8]. Palliative care is multi professional with recognition of the input of nurses, doctors, social workers, spiritual care providers and allied health professionals (where resources permit).

The cornerstone of palliative care is the relief of pain and other distressing symptoms- although palliative care cannot be said to be present if pain control is the only intervention available. My patient, in severe physical pain, is unable to accept the holistic approach until pain is relieved. Other dimensions of psychological and spiritual pain must also be assessed and managed.

The introduction of morphine is the focus of this paper, and is the success that enabled the provision of cheap and effective palliative care to those who are in severe pain. Previously there was no relief for these patients. Thus they were unable to think or make decisions, have autonomy, for themselves and their families. Once affordable oral morphine, suitable for use in the home is available, it is against justice for this treatment, prescribed at the hands of trained and legal prescribers, to be inhibited by bureaucratic channels in country.

## The founding of Hospice Africa Uganda

The introduction of this essential component of health care was a particular challenge in an African country such as Uganda in 1993. Uganda, which lacks economic resources, has few doctors (1: 19,000 population), and nurses (1:5,000 population) and where 57% of the population never see a health worker. Traditional healers are the first port of call in illness, and there is 1 traditional healer to 450 population. These traditional healers are holistic in their approach and culturally more acceptable to those in the village than Western medicine which can be expensive. Curative options are usually few for people who present with cancer- firstly they present very late, as they spend some time with the traditional healer, fear hospitals and have very little money. There is also only one source of radiotherapy and chemotherapy and that is in Kampala, far away from most Ugandan dwellings, and cannot meet the needs of all patients.

In 1993, Hospice Africa Uganda (HAU) was established [9,10], with the vision of facilitating the initiation and expansion of palliative care throughout Africa [11]. The aim was to create an affordable and culturally acceptable model in an African country, which could be adapted to the needs of other African countries. The Hospices and palliative care services in African countries would have an ethos to guide them to care for patient and family in this special time of life (although of course South Africa and Zimbabwe had an earlier history of the introduction of hospice). After a feasibility study of countries requesting such a service, Uganda was chosen because of the trust it had established in 1993 of the International communities, the great increase in cancers due to a high prevalence of HIV and the commitment of the Ministry of Health to support relief of the suffering of so many Ugandans by importing an affordable form of oral morphine which could be used in the home.

Hospice Africa Uganda commenced with very little funding, and began providing principally a home clinical service with the addition of a hospital consultation service. Working in partnership with the Ministry of Health commenced immediately. Teaching at Makerere University for undergraduate and post graduate doctors and for nurses in training was also initiated. Thus all doctors who have qualified to practice medicine since 1994 have been trained in palliative care. This year, the first unit of palliative care was commenced in Makerere University under Internal Medicine.

With the help of the Ministry of Health, oral affordable morphine was brought into Uganda in September 1993. This meant importing powder and making up the oral morphine using a formula perfected in Singapore during Dr Merriman's seven years there, and used for the first time in Africa in Nairobi Hospice 1990-2. Among the first patients, at the first Christmas, was a little boy of 7, named Bashir. He had had a forequarter amputation for osteosarcoma of the scapula. When he was referred to Hospice he already had cancer in secondary sites including the liver. He was in severe pain from the operation, and the skin grafts to the stump. His grandmother panicked when he developed malaria and she wanted to take him home to die. Granny having told hospital staff he lived 20 kms away, Dr Merriman, now alone as the little team had gone to their homes for Christmas, set out for home with a young interpreter from the ward caring for another patient. Thus they found that home was 60 kms away and the last 20 kms required them to transport him up a mountain! They managed to reach there in the donated 4WD, and then the team were committed to visiting him twice weekly until he died 6 weeks later. Thanks to the fact that he had morphine, he died a happy child and at peace. Without palliative care, and in particular without the availability of morphine to control his pain, this boy would have had no opportunity for a pain free and peaceful end to his life. His family would have suffered alongside him.

More than 16,000 patients and families have since received such care from Hospice Africa Uganda- and many more receive high quality palliative care from doctors, nurses, and other health professionals trained by HAU initially.

### Legislative change and specialist training to increase access

We realised that in order to make pain control a reality for all those who needed it, we had to increase the number of prescribers so that opioids could be accessed by patients across a range of health care settings and districts. In 2002, the Government agreed to change the statute. The revolutionary change enabled a new cadre of opioid prescribers: midwives were already allowed by statute to prescribe pethidine. To the same statute was added that nurses/clinical officers who had successfully undergone training at HAU for nine months and subsequently registered with the Ministry of Health, could prescribe morphine.

From 2000, due to a millennium grant from the British Royal Air Force, a similar programme of introducing advocacy for affordable morphine and training health professionals in parallel with morphine availability was rolled out initially to Tanzania and later to other countries. We were able to achieve this through a strategic advocacy approach to influence governments, health professionals, Universities and other health training institutions. Morphine is now available in 11 countries in Africa with the consent of their Governments. However, although there is a strong desire for professionals to be able to prescribe and control pain, and the training provides the skills to do this, experience has shown us that the provision of training is not enough to ensure that pain relief is delivered. Health professionals need the subsequent support and mentoring of someone with African experience to work side by side with them, to introduce record keeping, monitoring and evaluation, organisational development and all those aspects of a sustainable organisation relevant to, and affordable in, their own unique circumstances.

Less than a quarter of African countries have now affordable oral morphine available. To address this problem, a combination of advocacy, education and training is required to ensure and maintain good analgesia for those in need. Again, training is vital and needs to come from African success stories. We require models of care and drug options that are relevant to the local context- and evidence-based palliative care needs to be founded on research and practice that has been shown to be effective and feasible in African settings.

## Morphine and end of life care in the home: Hospice Africa's experience

### Methods of delivery

Patients in Africa prefer to die in their own homes [12-14]. Our focus has been on critical care and end of life care in the home since 1993. However, we do attend hospitals on consultation and subsequently follow the patients up at home.

**Figure 1:**
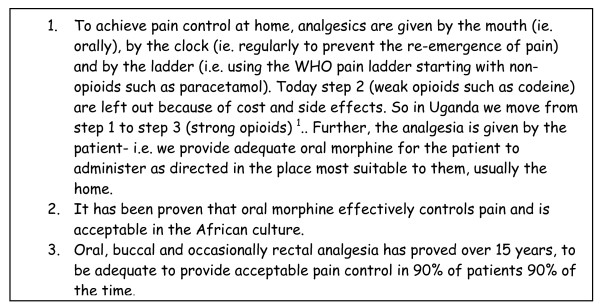
**Principles of pain relief**.

For pain relief to be possible continuously in the home, morphine needs to be given orally. The patient and family are trained in giving morphine at regular intervals so that the pain does not return. They are instructed in giving an extra dose for breakthrough pain and the number of extra doses used, are calculated to adjust the daily dose at the next visit of the team (Figure 1).

### Which route of administration?

In 1993, we arrived with a donation of 5 syringe drivers for parenteral morphine. We used them 3 times the first year, twice the second and never since.

Firstly we can effectively manage pain with oral morphine. If the problems of high or low intestinal obstruction occur (obstruction from cancer of the oesophagus, relatively common and situated high) or ovary (not so common and lower down according to the site of the metastases), we then use concentrated morphine dripped into the mouth and this is absorbed from the buccal mucosa with good effect. Occasionally we use MST (slow release morphine tablets) rectally, but this is expensive.

### Managing opioid side effects

Providing a laxative for the anticipated constipation has proved a challenge as the laxative of choice, sennakot is expensive and not always available. Bisacodyl has greater availability at a cost. We are now prescribing the seeds of paw paw, dried and crushed, one to five teaspoons at night. This gives very good results and paw paw fruits are readily available [see Additional file 1]. Then we turn to the margarine for the high or low impaction. One dessertspoon taken before breakfast lubricates impacted stool. We manage most constipation with this regime while continuing with paw paw seeds and occasionally adding in bisacodyl.

### Neuropathic and bone pain

Morphine is not the treatment of choice for all pains, and neuropathic pain and bone pain are treated by conventional means, usually antidepressants or anti epileptic medications for neuropathic pain not responding to oral morphine and non steroidal anti inflammatory drugs (NSAIDS) for bone pain [15]. Our choice of medication is limited by cost and availability, but we do relieve most pains. Some of the medications usually prescribed for HIV neuropathic pain such as gabapentin are prohibitively expensive for use in our setting. To manage neuropathic pain of herpes zoster, we are now using the milk of the frangipani tree [16], [see Additional file 2]. This is applied three times a day to the affected dermatomes, before the vesicles appear and later for post herpetic neuralgia (see reference [17]).

## Lessons from morphine production in Uganda

The process that has been established for the safe use of opioids for home palliative care in Uganda is as follows.

1. Import morphine powder from supplier: appropriate paper work and Government acceptance of the requirements for the country taken into account through the National Drug Authority.

2. Morphine powder is transferred to the dispenser. The dispenser is overseen by a registered pharmacist.

3. Morphine is made up as follows [17]:

a) Equipment required:

1. an accurate scale (usually an electronic scale capable of weighing tiny amounts of powder)

2. Ability to boil and filter water

b) Powder is weighed and mixed with boiled, filtered water [see Additional file 3].

c) The preservative, bronopol is added and the solution diluted with the required amount of water to make it in the following strengths:

1 mg per ml,

10 mgs per ml and

20 mg per ml.

d) Each strength is colour coded: green for 1 mg per ml, pink for 10 mgs per ml and blue for 20 mgs per ml. The colour is cake colouring.

4. The liquid is now poured into recycled plastic water bottles. These bottles are washed carefully, in the pharmacy, before use. The patient is also provided with a 5 ml syringe without a needle. These are soon to be replaced with cups because of the moulded needles to syringes now being produced. With the syringe, they are shown how to draw it up, by pouring the liquid into the lid of the bottle, which holds about 5 mls. With the cups it will be poured in directly and measured.

5. The patient or relative receives the morphine from the dispenser. The person who collects signs the Controlled Drugs book.

The formula for oral morphine, as used in Hospice Africa Uganda, is found in the "Blue Book" (see reference [17]).

Patients and families are given a leaflet with instructions to keep the medicine out of the reach of children. The leaflet also explains about the use of morphine. It is in the local language as well as English. The patient and relative are asked to return any unused morphine, and if this is not possible because of distance, to destroy it (e.g. put it down pit latrine) and to note how much they destroyed.

The cost of morphine in Uganda is low. 500 mgs of morphine diluted to 500 mls is the cost of a loaf of bread. The average dose controlling pain in our patients is 30 mgs per day. Therefore, with morphine 5 mgs being taken 4 hourly and a double dose at night, the bottle lasts the average patient 10 days allowing for break through doses of the regular 4 hourly dose if required.

## Replicating our successes in other African Countries

This formula and our training is now being carried to countries where Governments have agreed to import powdered morphine. Advocacy to Governments, medical schools and training schools is bringing pain control to more and more patients, but there is still a huge need.

Critical illness in the home and end of life care is being provided in a small number of countries by hospice and palliative care organisations. HAU has shown how commencing from very little, with the support of Government and networking organisations, pain control opens the door to providing holistic care to the patient and family. The evaluation of our public health approach to expanding the availability of morphine has demonstrated that any fears of opioids being diverted from health care into the hands of recreational users are unfounded, and that systemic analysis of the opioids supply chain is essential to unblock the system [18]. However, we also identified that there are problems as there are many steps to be taken to make this effective, with many rules and regulations which prevent our patients getting acceptable pain control in some of the Government health systems.

We have identified a few challenges in the replication of our Ugandan successes in other countries. These are observed in variation in price, in the way patients are instructed and reinforcing myths about addiction, in reinventing the wheel for the right formulae, strengths and storage. These delays exhibit maleficence. There is physical harm to the patient, suffering for the patient and family.

In visiting other African countries, we recently found that the cost of morphine in one was 5 times that in Uganda, posing a significant challenge to models of affordable pain management and palliative care [19]. In another country, a pharmacist was questioning each patient or relative at the pharmacy as to why they were taking an addictive drug and reducing the dose if he anticipated a stock out. Another pharmacist in a Muslim country was using a formula made up with alcohol as preservative when this is not acceptable to the Muslim. There is an urgent ethical need for those of us who have experienced success to share experiences with those initiating care and facing challenges to provide sustainable, affordable and effective models of care.

## Conclusion

Getting the system right so that morphine is available and reaches those in need is the beginning of the holistic approach. There are many other pains beside physical pain, and that may be as distressing: psychological, spiritual and social problems, which must be addressed. We have focused here on opioids as a specific challenge, but the effective management of these other problems also require urgent attention. Effective analgesia and the availability of opioids is essential to allow us to then approach the range of holistic needs of a patient who is in severe pain and has a terminal prognosis. Hospice Africa, together with other established palliative care services and networking with the more recently established regional advocacy organisation, the African Palliative Care Association, are working together to increase the coverage, and ensure quality, throughout Africa for those in need.

Oral morphine, made up near to the patients in need, is now recognised as the most affordable and effective way to control severe pain for cancer and AIDS, in the African situation. Having proved that this affordable method works, we, who witness the terrible suffering still going on in Africa from cancer, AIDS and other diseases, are ethically bound to try and make it reach all in need in Africa. Only thus can we bring dignity of life and death [20], based on the four pillars of ethics, to those in need.

Working with limited resources and in a different cultural setting, certain observations have been made which might, with further research, assist pain control methods and palliative care in more developed countries.

## Competing interests

The authors declare that they have no competing interests.

## Authors' contributions

AM is the invited author and conceived and wrote the original paper.

RH edited, contributed advice and further references.

All authors read and approved the final manuscript.

## Supplementary Material

Additional file 1 **paw paw (papaya) fruit whose seeds are dried, then crushed to be used to anticipate constipation from morphine use**. Click here for file

Additional file 2 **The frangi pani tree, available in most tropical climates, produces a milk when a twig is snapped off, which when collected and applied to the affected area of herpes zoster, paralyses sensory fibres and controls the neuropathic pain for 8 hours**. Click here for file

Additional file 3 **Peter Mikajo, Dispenser at HAU, prepares morphine powder before weighing while making up liquid morphine**. Click here for file
