# Supplementary figures and images for: Pain Control in the African Context: the Ugandan introduction of affordable morphine to relieve suffering at the end of life
Source: Philos Ethics Humanit Med. 2010 Jul 8;5:10. doi: 10.1186/1747-5341-5-10 (PMC2914675; doi:10.1186/1747-5341-5-10)

## Slide 1
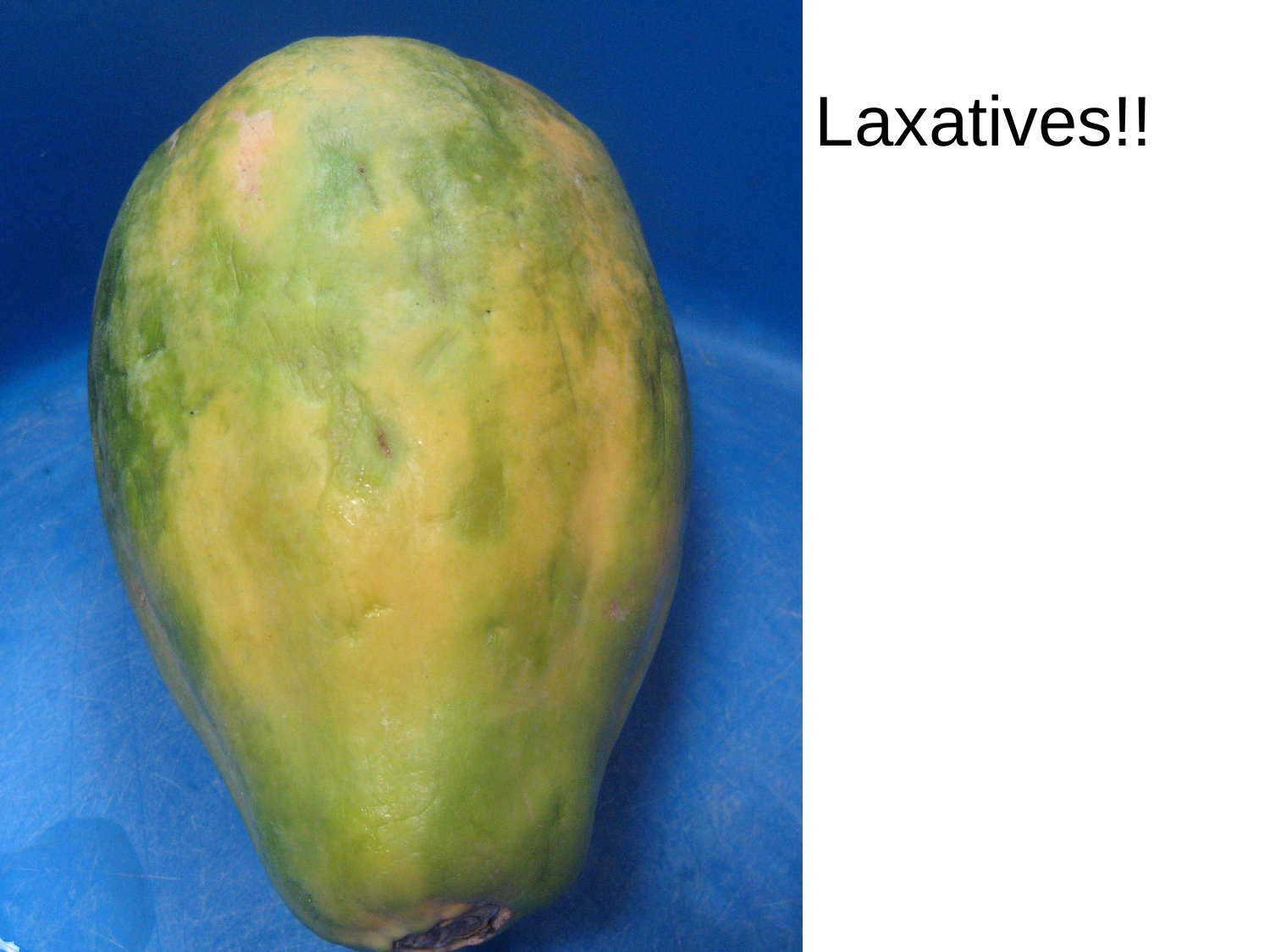

# Laxatives!!

## Slide 2
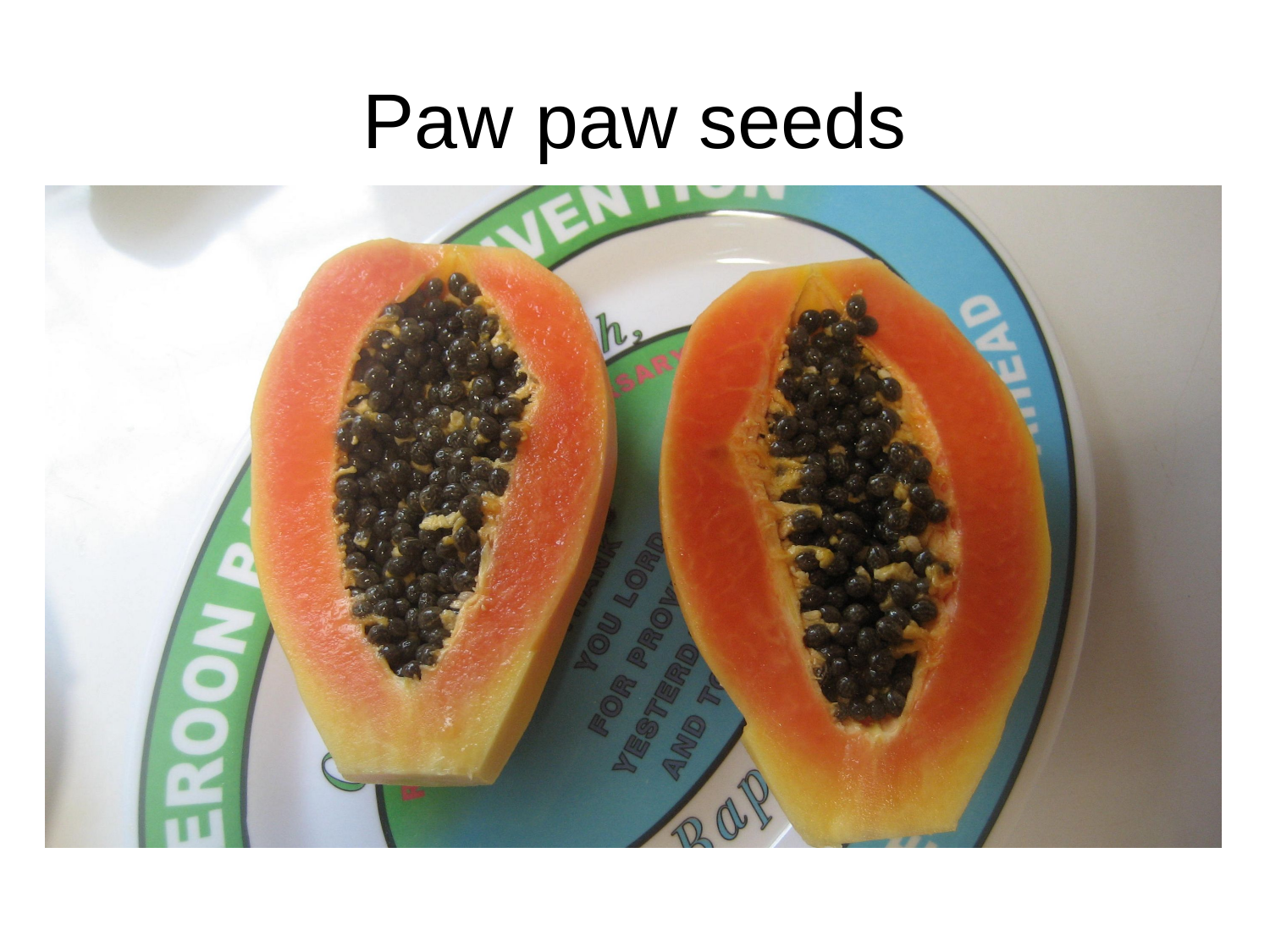

# Paw paw seeds

Supplement: Additional file 1 — paw paw (papaya) fruit whose seeds are dried, then crushed to be used to anticipate constipation from morphine use. [file 1747-5341-5-10-S1.PPT]

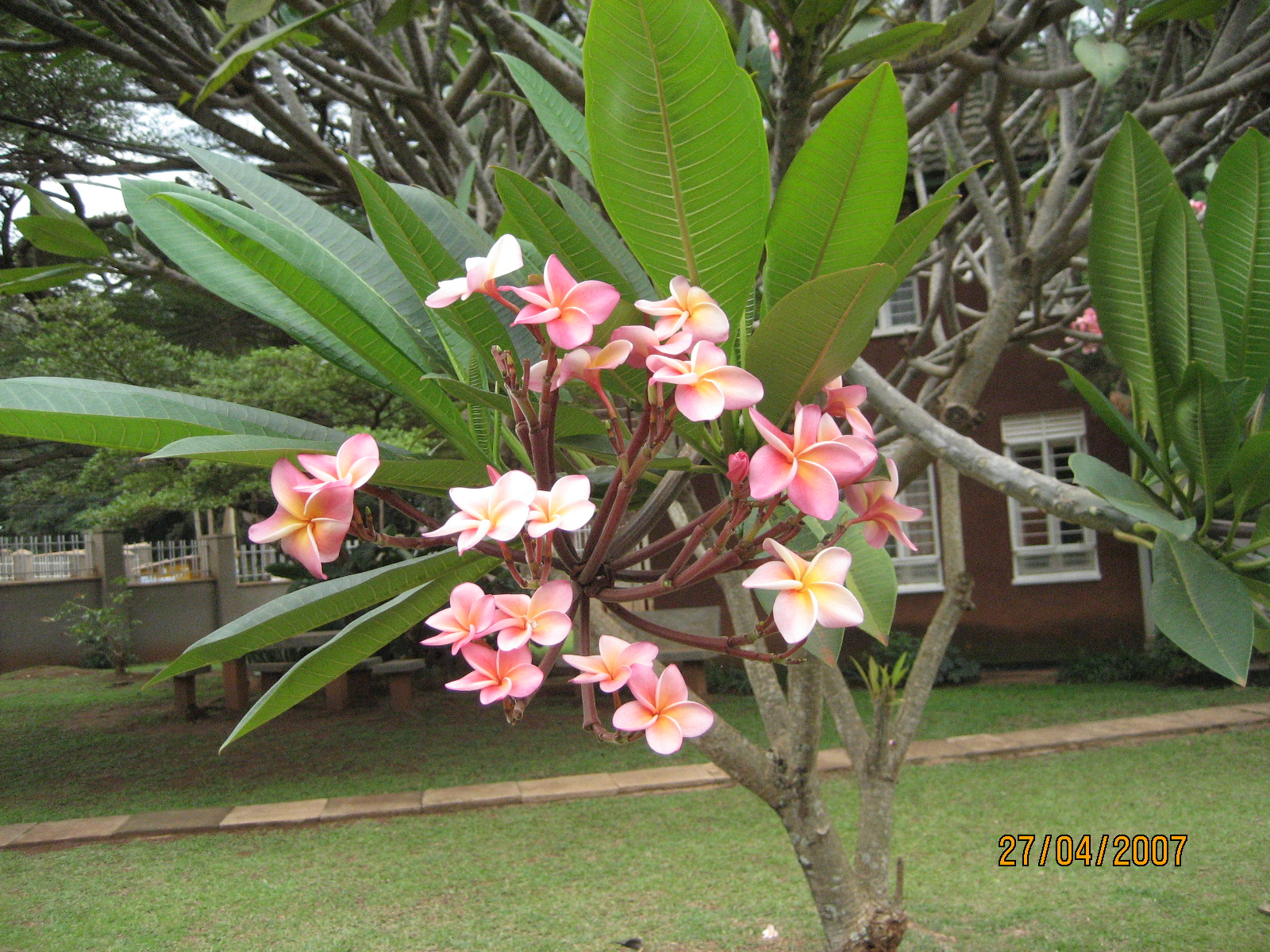

Supplement: Additional file 2 — The frangi pani tree, available in most tropical climates, produces a milk when a twig is snapped off, which when collected and applied to the affected area of herpes zoster, paralyses sensory fibres and controls the neuropathic pain for 8 hours. [file 1747-5341-5-10-S2.JPEG]

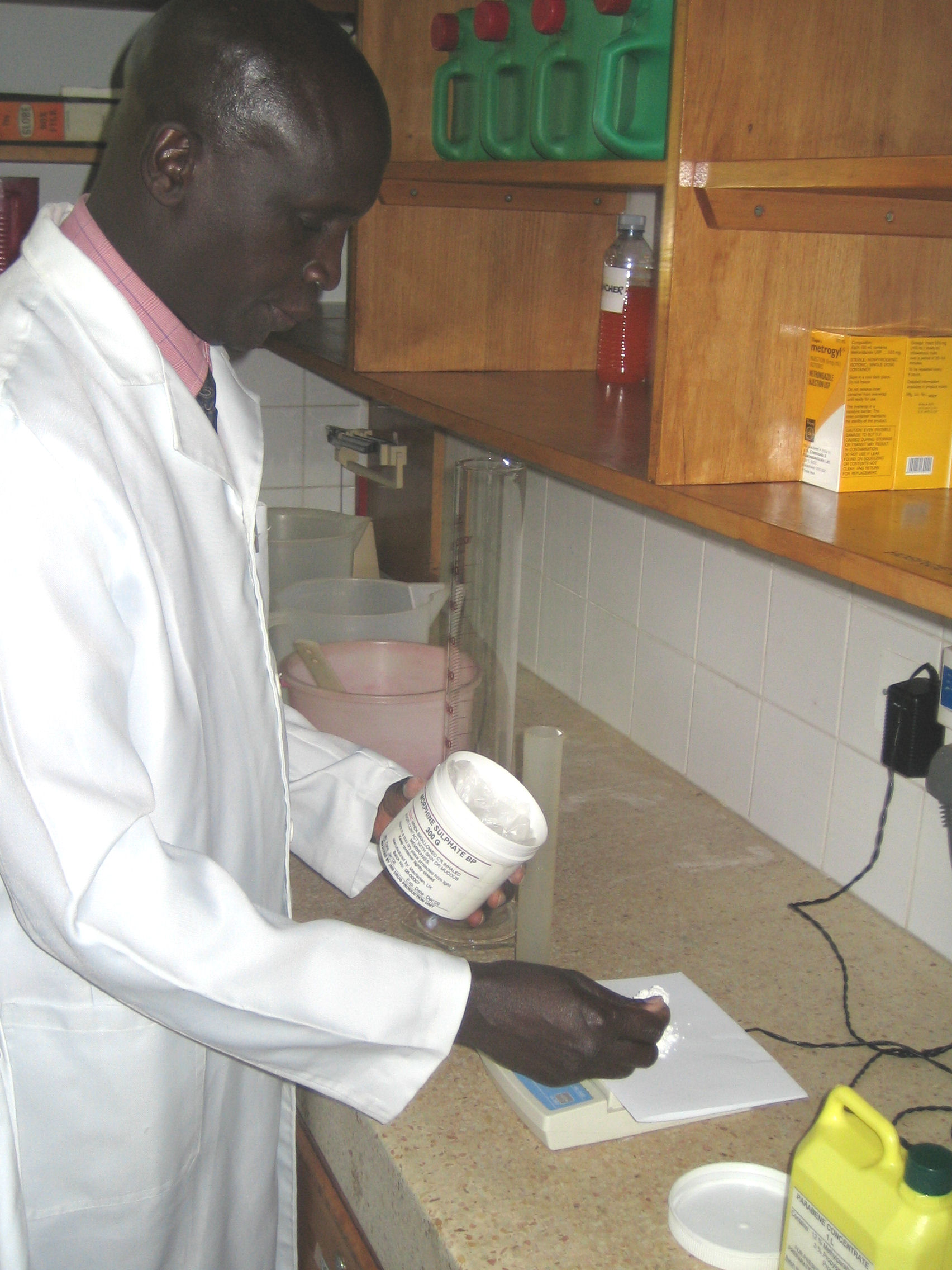

Supplement: Additional file 3 — Peter Mikajo, Dispenser at HAU, prepares morphine powder before weighing while making up liquid morphine. [file 1747-5341-5-10-S3.JPEG]
